# Spinal Cord Compression Secondary to Benign Metastasizing Leiomyoma

**DOI:** 10.7759/cureus.21845

**Published:** 2022-02-02

**Authors:** André Ferreira, Mariana Malheiro, Ana Martins

**Affiliations:** 1 Medical Oncology, Hospital de São Francisco Xavier, Lisbon, PRT; 2 Medical Oncology, Hospital CUF Tejo, Lisbon, PRT; 3 Medical Oncology, Centro Hospitalar de Lisboa Ocidental, Lisbon, PRT

**Keywords:** gynecologic malignancies, vertebrectomy, spinal cord compression, lung metastasis, benign metastasizing leiomyoma

## Abstract

Benign metastasizing leiomyoma is an extremely rare disease characterized by the presence of extrauterine spread of smooth muscle cells with histological, molecular, and immunological patterns similar to those of benign uterine leiomyomas. Benign metastasizing leiomyoma is often asymptomatic, and it presents as an incidental radiology finding of well-defined multiple pulmonary nodules with varying sizes. It is more frequent in premenopausal women, and a previous history of uterine leiomyomas resected in the past is found in most of the cases. There are very few case reports of benign metastasizing leiomyoma causing spinal cord compression. The authors report an uncommon case of a premenopausal woman with spinal cord compression one year after the diagnosis of benign metastasizing leiomyoma to the lung. Given that spinal cord compression is an oncologic neurosurgical emergency, rapid diagnosis and management are essential to prevent irreversible neurological deficits.

## Introduction

Uterine leiomyoma is the most common gynecologic tumor in women of reproductive age [1], affecting 20% to 30% of those older than 35 years [2]. It consists of uterine cells with smooth muscle differentiation and spindle-like features. It is considered to be benign histopathology, exhibiting low mitotic activity, lack of anaplasia and necrosis, and limited vascularization [3]. Despite these benign features, leiomyomas can rarely spread to extrauterine sites, predominantly to the lungs. Benign metastasizing leiomyoma (BML) tumors of the lungs are bilateral in 70% of cases, with a mean number of nodules of approximately six, and a mean nodular size of 1.8 cm [4]. Other sites of less frequent metastasis include bone, spine, lymph nodes, retroperitoneum, and intravascular spread [4]. It is thought to originate from the hematogenous metastasis of myoma cells following myomectomy [5]. The clinical course of BML is usually asymptomatic but can present as a rapidly progressive disease that can lead to respiratory failure and death. Although there is no established standard treatment for BML, treatments are based on endocrine therapy plus surgical or medical oophorectomy, given the hormone-sensitive characteristics of BML [6].

Spinal cord compression (SCC) is a devastating but potentially treatable emergency. It can be caused by trauma, tumor, epidural abscess, and epidural hematoma [7]. The most common symptoms are aching back pain, numbness, cramping or weakness in the arms or legs, and loss of bowel or bladder control. Spinal metastases are common in cancer, but they cause SCC only when they extend from the bone into the epidural space [7]. The most frequent causes of metastasis to the vertebral column are breast, prostate, and lung cancers [8].

## Case presentation

**Figure 1:**
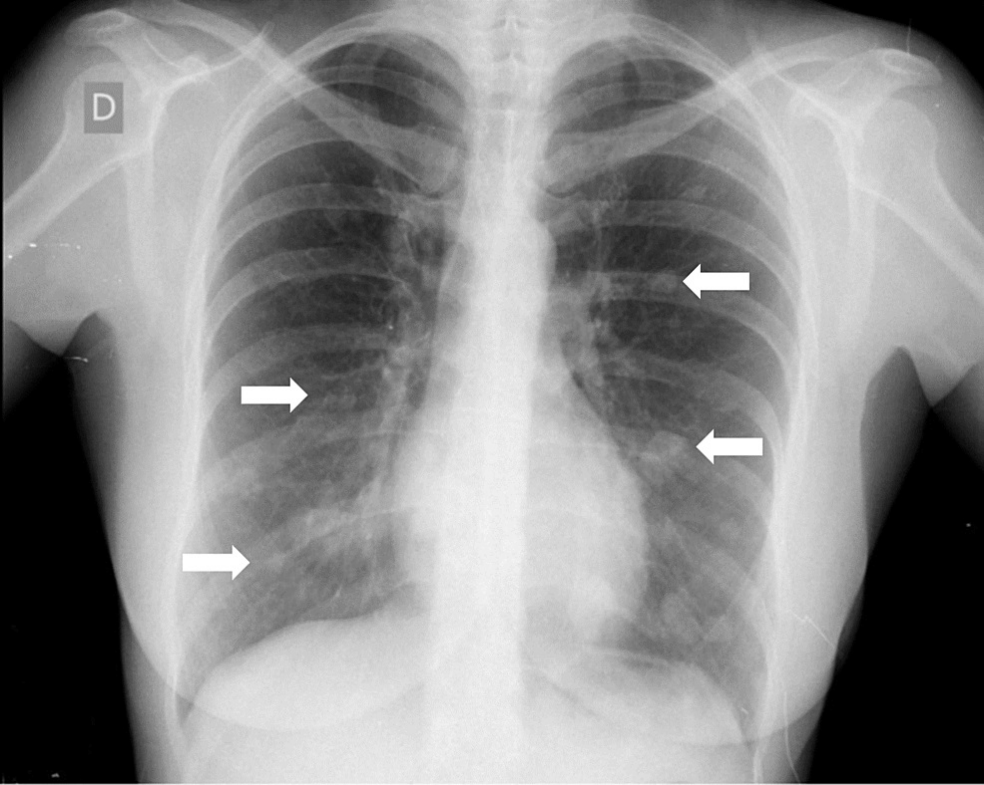
Chest radiograph showing bilateral pulmonary nodules (arrows)

We present a case of a 42-year-old woman, active smoker (10-pack-years), who was referred, in August 2017, by her general practitioner to a pulmonology outpatient clinic for further evaluation of a routine chest radiograph, showing multiple nodules of variable size in both lung fields (Figure 1).

Of the relevant past medical history, she had asthma and generalized anxiety. She was submitted to uterine myoma resection in 2011 and to hysterectomy due to uterine leiomyoma in 2014. She did not have any previous pulmonary disease, such as pulmonary tuberculosis or pneumonia. There was no history of chest symptoms, cough, dyspnea, chest pain, fever, weight loss, or night sweats. On physical examination, she had no significant changes.

**Figure 2:**
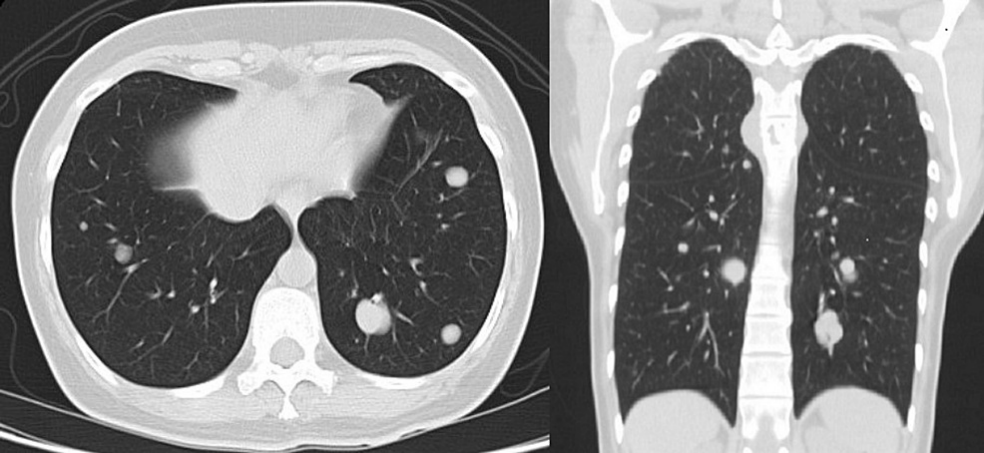
Chest CT with transverse and coronal slices demonstrating multiple heterogeneous nodules

Chest CT (September 2017) revealed multiple nodules in both lungs with a maximum diameter of 30 mm, located peripherally and centrally (Figure 2). The flexible bronchoscopy with biopsy showed infiltration at the level of one of the segments of the basal pyramid and signs of chronic bronchitis. The culture from bronchial secretions and bronchial brush was negative. Histology of the fragments of bronchial mucosa had no dysplasia or neoplastic infiltration. Positron emission tomography-computed tomography (PET-CT) was performed to exclude other places of possible malignant primary disease. There was no abnormal fluorodeoxyglucose (FDG) uptake within the multiple suspicious nodules. The guided gynecological, thyroid, renal, and abdominopelvic studies were all normal. For diagnostic purposes, the patient was then submitted to resection of two nodules in the superior and medium right lobes through video-assisted thoracoscopic surgery (VATS) in January 2018. Positivity for α-smooth muscle actin and desmin, along with 100% positive of estrogen and progesterone receptors, were consistent with pathologic diagnosis of benign metastasizing leiomyoma.

She was referred to an oncology unit in March 2018 and was treated with ovarian function suppression with monthly goserelin.

Due to the frequent non-attendance of the patient at the oncology consultation, no imaging was performed.

In October 2018, she went to the emergency department with disabling axial back pain, with anterior radiation from the left, resistant to analgesia, and accompanied by a sensation of numbness in the lower limbs and abdomen, with an associated decrease in muscle strength, which made walking difficult. On neurological examination, she presented paraparesis with muscular strength grade 4 in lower limbs, with sensitive level in T6.

**Figure 3:**
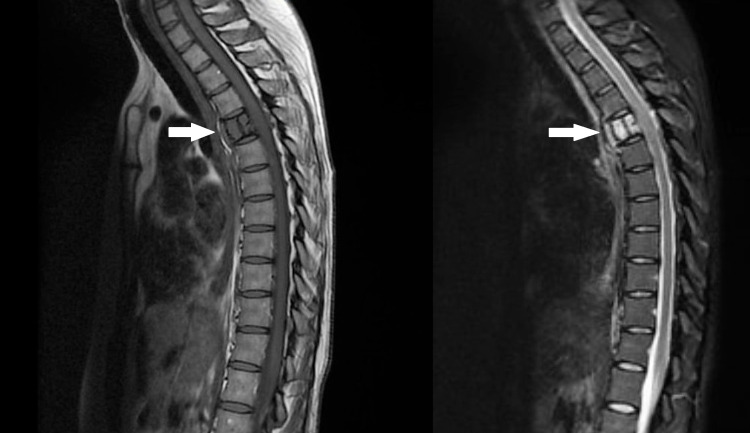
MRI of the spinal column showing a lesion centered on the vertebral body of T5 (arrows)

She performed a magnetic resonance image of the spinal column that showed a T1-hypointense and a T2/Short-TI Inversion Recovery (STIR)-hyperintense lesion centered on the vertebral body of T5, with exuberant enhancement after gadolinium administration, associated with a circumferential epidural lesional component at this level, conditioning spinal cord compression (Figure 3).

She underwent a T5 vertebrectomy with the application of an expandable vertebral body cage and T3-T4-T6-T7 transpedicular fixation.

**Figure 4:**
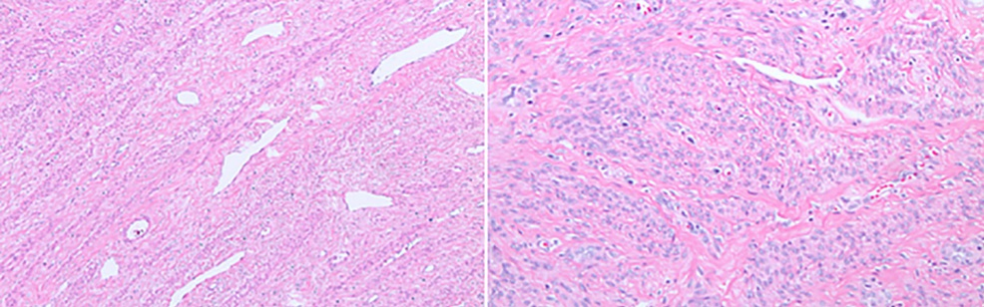
Vertebral mass histopathology demonstrating the typical morphology of a leiomyoma with fascicular pattern of smooth muscle cells

Bone histopathology revealed bone infiltration of malignant cells positives for smooth muscle actin, desmin, and caldesmon and negative for S-100, CK AE1/AE3, and CAM-5.2 (Figure 4).

After the multidisciplinary discussion of the case with the oncology department, she underwent laparoscopic bilateral salpingo-oophorectomy for disease control. She also started tamoxifen due to disease progression under goserelin.

After the vertebrectomy, the patient reported back pain without motor and sensory deficits. She started pregabalin with progressive improvement of pain symptoms.

The patient maintains hormone therapy with tamoxifen and has done a follow-up in oncology, neurosurgery, and gynecology departments, without evidence of disease progression or new neurological symptoms.

## Discussion

BML is a rare disease characterized by well-differentiated smooth muscle cells derived from uterine leiomyomas presenting in extra-uterine sites, namely lungs (79,5%), lymph nodes, abdominal and pelvic cavity, nervous system, and bone [9]. BML It is most often found in premenopausal women with a known history of uterine leiomyoma resection or hysterectomy, making BML a condition that presents an interesting diagnostic and therapeutic challenge given its rarity and indolent nature [10]. Immunohistochemically, BML presents with smooth muscle markers (smooth muscle actin and desmin) along with being estrogen and progesterone positive [11]. Spine metastases from BML have only been reported in a few case report articles.

Acute compression of the spinal cord is a devastating but treatable disorder. The patient presented with neurological symptoms suggestive of SCC, such as pain and motor and sensory deficits in the lower limbs. The MRI confirmed the diagnosis of SCC at the level of T5. The patient underwent an urgent vertebrectomy, given that spinal surgery is the most rapid method for relief of acute spinal cord compression, when feasible.

Due to disease progression under goserelin - a luteinizing hormone-releasing hormone agonist - and because BML is a hormone-sensitive disease, the patient was discussed in a multidisciplinary team, and it was decided to undergo laparoscopic bilateral salpingo-oophorectomy and start endocrine therapy with tamoxifen - a selective estrogen receptor modulator - for disease control.

## Conclusions

BML is a rare cause of extrapelvic metastases, predominantly to the lungs, usually secondary to gynecological instrumentation. For this reason, the multidisciplinary approach of BML is fundamental between oncologists and other medical specialties for adequate evaluation and management since there is not yet a consensus on treatment strategy. Our case represents a serious complication of BML in a premenopausal woman many years after uterine myoma resection and hysterectomy, given that acute spinal cord compression is a medical emergency that requires swift diagnosis and treatment to prevent irreversible spinal cord injury and long-term disability. In conclusion, although this disease contains the name "benign," patients should be followed for potential serious disease complications.
